# Indirect Co-Culture with Tendons or Tenocytes Can Program Amniotic Epithelial Cells towards Stepwise Tenogenic Differentiation

**DOI:** 10.1371/journal.pone.0030974

**Published:** 2012-02-10

**Authors:** Barbara Barboni, Valentina Curini, Valentina Russo, Annunziata Mauro, Oriana Di Giacinto, Marco Marchisio, Melissa Alfonsi, Mauro Mattioli

**Affiliations:** 1 Department of Comparative Biomedical Science, University of Teramo, Teramo, Italy; 2 Department of Biomorphology, University of Chieti, Chieti, Italy; 3 Department of Biomedical Sciences, University of Chieti, Chieti, Italy; 4 StemTeCh Group, Chieti, Italy; University of California, Merced, United States of America

## Abstract

**Background:**

Amniotic epithelial cells (AEC) have potential applications in cell-based therapy. Thus far their ability to differentiate into tenocytes has not been investigated although a cell source providing a large supply of tenocytes remains a priority target of regenerative medicine in order to respond to the poor self-repair capability of adult tendons. Starting from this premise, the present research has been designed firstly to verify whether the co-culture with adult primary tenocytes could be exploited in order to induce tenogenic differentiation in AEC, as previously demonstrated in mesenchymal stem cells. Since the co-culture systems inducing cell differentiation takes advantage of specific soluble paracrine factors released by tenocytes, the research has been then addressed to study whether the co-culture could be improved by making use of the different cell populations present within tendon explants or of the high regenerative properties of fetal derived cell/tissue.

**Methodology/Principal Findings:**

Freshly isolated AEC, obtained from ovine fetuses at mid-gestation, were co-incubated with explanted tendons or primary tenocytes obtained from fetal or adult calcaneal tendons. The morphological and functional analysis indicated that AEC possessed tenogenic differentiation potential. However, only AEC exposed to fetal-derived cell/tissues developed *in vitro* tendon-like three dimensional structures with an expression profile of matrix (*COL1* and *THSB4*) and mesenchymal/tendon related genes (*TNM*, *OCN* and *SCXB*) similar to that recorded in native ovine tendons. The tendon-like structures displayed high levels of organization as documented by the cell morphology, the newly deposited matrix enriched in COL1 and widespread expression of gap junction proteins (Connexin 32 and 43).

**Conclusions/Significance:**

The co-culture system improves its efficiency in promoting AEC differentiation by exploiting the inductive tenogenic soluble factors released by fetal tendon cells or explants. The co-cultural system can be proposed as a low cost and easy technique to engineer tendon for biological study and cell therapy approach.

## Introduction

Tendon overuse injuries, which are referred to as tendinopathy affect millions of people in occupational and sport settings [1], [2]. Until the present time, pharmacological (anti-inflammatory) and non-pharmacological therapies (physiotherapy and surgical intervention) provide exclusively symptomatic relief [3]–[5] since an effective treatment able to stimulate a complete process of tendon healing remains to be developed. Biologically based strategies able to stimulate cells activity and extracellular matrix deposition have received increasing attention [3]. In this context, stem cell-based therapy seems to represent the most promising frontier to restore tendon function [3], [6]–[8]. Spontaneous healing is generally incomplete and frequently leads into fibrosis, thus, compromising the biomechanical properties of tendon causing significant dysfunction and disability. Although the mechanism involved in this failure remains still unknown, it probably resides in the high degree of differentiation of this tissue and in the low cellularity that limits the capacity of differentiated tenocytes to replicate in adult organisms [3], [9]. For this reason, cell-based therapy or tissue engineering were suggested as an ideal approach to support tendon healing [3], [10], and to this aim several cell types have been used under preclinical settings [8].

Autologous tenocytes seem to be the more appropriate cells for their innate ability to develop the highly organized tendon extracellular matrix in the absence of any risk of immune-rejection. However, the few tenocytes harvested from adult tissue and the high morbidity of the donor site have severely limited the use of autologous cells [3].

Stem/progenitor cells have been alternatively proposed for a direct transplantation or after a previous *in vitro* differentiation into tenocyte-like lineage [8]. Mesenchymal stem cells (MSCs) have been the most largely used [11], [12] for this purpose, cause their low ability to integrate within tendon host tissue, a transgenic transformation was adopted [8]. Until now, different transgenic approaches have been proposed either to up regulate growth factors involved in tendon repair [13], [14], or gene expression controlling tenocyte-lineage differentiation [15], [16].

An efficient tenogenic differentiation protocol has recently been proposed for MSCs based on their co-culture with primary tenocytes [17]–[19]. The co-incubation with differentiated cell lines as osteoblast [20], chondrocytes [21], and tendon-ligament cells [17]–[19] seems, in fact, to commit stem/progenitor cells versus the relative cell lineage phenotype. The co-culture systems involved the direct cell-cell interaction [19] or the use of cell-specific conditioned medium [17], [18]. This last approach appears to be particular attractive since it operates under the stimulatory influences of paracrine humoral factors released by appropriate cell types, thus, allowing a low cost process of *in vitro* differentiation eliminating any undesirable risk of cell contamination. The co-culture systems, in addition, reproducing the specific microenvironment that physiologically regulate cell development, tissue maintenance, and regeneration may represent also a valid tool to test *in vitro* the ability of stem/progenitor cells to undergo tissue specific differentiation before attempting the site-directed delivery into healthy or injured tissues.

Starting from these premises, the present research was designed to verify whether the co-culture could be applied to epithelial derived amniotic cells (AEC), and whether the efficiency of the co-cultural system could be improved by using fetal samples, with higher regenerative properties, or tissue explants co-cultures.

To these aims, AEC immediately after isolation were co-cultured in the presence of adult *vs.* fetal tendon explants or, alternatively, with the respective primary derived cells (fetal or adult tenocytes). The tenogenic inductive potential of different co-cultures were compared under the 28 days of incubation (7, 14, 28 days) by analyzing cell phenotype, cell proliferation index and by monitoring the *in vitro* molecular AEC reprogram versus tendon lineage tissue.

The present results indicate that all tendon derived samples (tissue explants and tenocytes) are able to generate a favorable microenvironment for tenogenic differentiation. Co-cultured AEC, in fact, stepwise differentiated to tenogenic lineage evolving through a mesenchymal transition phase. Interestingly, tenocytes and explants derived from the fetus possessed the highest tenogenic differentiative properties that led AEC to form *in vitro* tendon-like structures with a more mature morphological organization and molecular profile. Altogether, these data suggest that AEC combined with co-culture may provide a low cost and large supply of tenocytes and/or engineered tendons to develop novel cell-based therapy.

## Results

### AEC characterization

**Figure 1:**
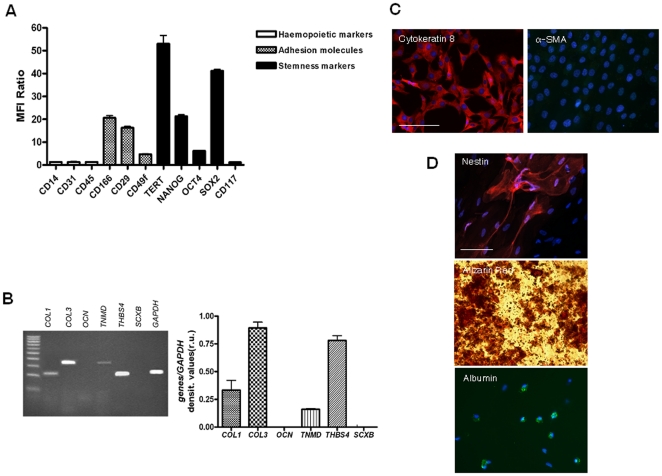
Molecular characterization of freshly isolated AEC. **A**) Levels of surface and intracellular stemness markers analyzed by flow cytometry and expressed as Mean Fluorescence Intensity (MFI) ratio. **B**) The mRNA content of tendon-related genes analyzed by RT-PCR. The bars show the standard error calculated on 3 independent experiments. **C**) Two representative images of cytokeratin 8 (epithelial marker) and α-SMA (mesenchymal marker) proteins detected in AEC by using an immunocytochemistry approach. The images show the blue nuclei counterstained with DAPI, and both proteins in red (Cy3). Scale bar for all images = 50 µm. **D**) The *in vitro* differentiation of AEC into endoderm (liver: bottom image) and ectoderm (neural cells: top image) cell lineages are documented by the immunocytochemistry detection of nestin and albumin, respectively. Nuclei were counterstained with DAPI. The mesodermal osteogenic *in vitro* differentiation (central image) was documented by the Alizarin Red staining. Scale bar for all images = 50 µm.

The enzymatic digestion of amniotic membranes allowed to isolate a purified AEC population as documented by the cytokeratin 8 expression and the molecular profile indicated by the flow cytometry (Fig. 1). In detail, freshly isolated AEC did not display any haemopoietic markers (CD14, CD58, CD31 e CD45). On the contrary, the cells expressed several surface adhesion molecules (CD29, CD49f and CD166), and the stemness markers TERT, SOX2, OCT4 and NANOG, while the CD117 resulted not expressed.

RT-PCR indicated that AEC clearly expressed Collagen type 3 (*COL3*) and Trombospondin 4 (*THBS4*), lower levels of Collagen type 1 (*COL1*), and Tenomodulin (*TNMD*), while did not express Scleraxis B (*SCXB*) and Osteocalcin (*OCN*).

Freshly isolated ovine AEC showed the ability to differentiate into cells from all three germ layers (Fig. 1). Endodermal lineage differentiation was obtained by incubating AEC under hepatic conditions. Round cells expressing the hepatic marker albumin were recorded after 10 days of culture. Analogously, the cells cultured in an osteoinductive medium showed an intense extracellular matrix mineralization described by Alizarin red staining.

Finally, AEC expressed the nestin marker when incubated in the presence of a neurogenic agent, the trans-retinoic acid. The differentiation towards neural cells was also suggested by the typical elongated cell morphology assumed by ∼15% of nestin positive cells.

### AEC morphology and activity were influenced by co-culture systems

The AEC cultured in α-MEM containing 10% FCS and without any growth factors (Table 1) proliferated with a medium doubling time of ∼38 hours during the first week of incubation reaching confluence in ∼3 weeks.

**Table 1:** Proliferation activity recorded in AEC maintained in culture for 28 days alone or in co-incubation with tenocytes and tendon explants derived from fetal or adult calcaneal tendons.

Co-cultural systems	Doubling time (hours)	Proliferation index (%)	
	Day 7	Day 14	Day 28
**AEC**	38±4.2	38±4.4	6±0.9
**AEC plus fetal:**			
Tenocytes	26±1.6*	32±3.8	29±3.2^a^
Explants	23±0.9*	40±4.6	38±4.3^b^
**AEC plus adult:**			
Tenocytes	29±4.8	12±2.1^b^	8±1.5
Explants	33±3.4	19±2.3^b^	23±3.2^a^

The doubling time was expressed as media ± SD of 3 replicates obtained from at least 3 different experiments. The data were compared by One Way ANOVA test followed by *post-hoc* Tukey test.

*Asterisk indicates values significantly different within the column (*p*<0.05).

The proliferation indexes are expressed as the % of Ki-67 positive cells/total cells recorded in 3 replicates obtained from at least 3 different experiments/group. The data were compared by One Way ANOVA test followed by *post-hoc* Tukey test.

a,b Different superscripts indicate values significantly different within each column (*p*<0.05).

The proliferation index analyzed from the second week of incubation progressively dropped passing from ∼40% to ∼5% at day 28 (Table 1).

**Figure 2:**
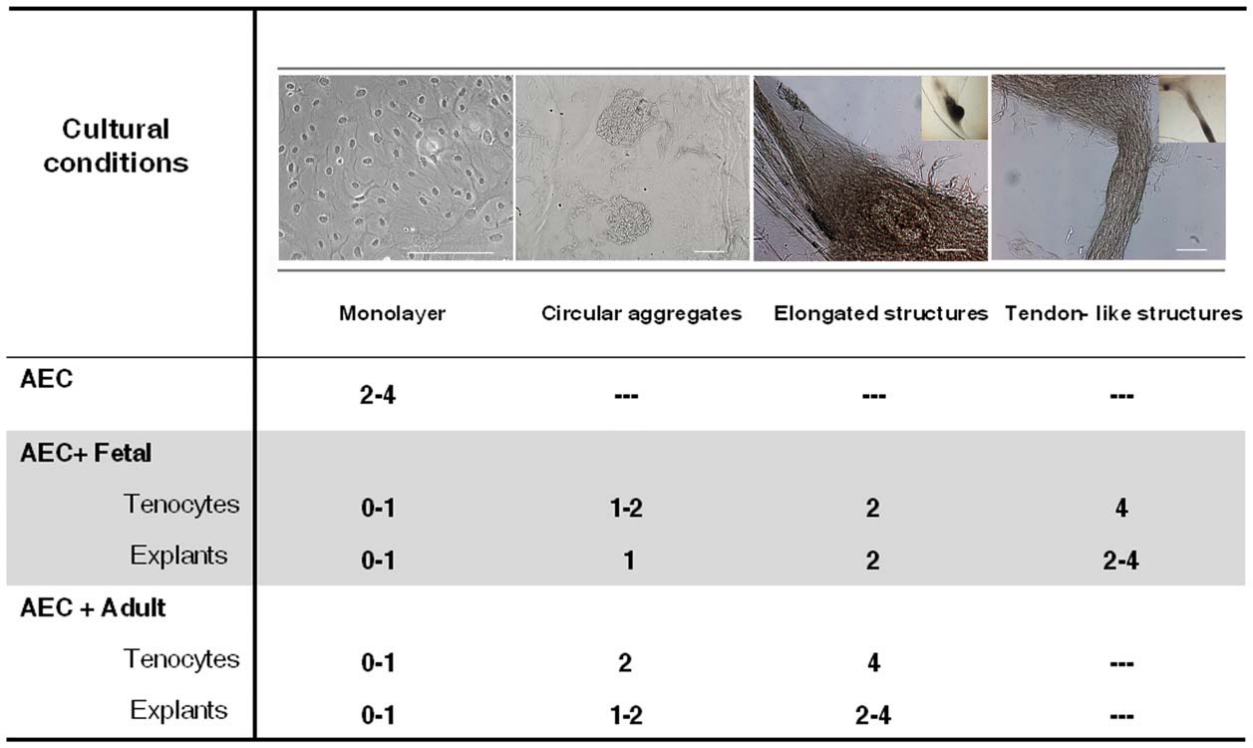
Morphology of differentiated AEC toward tenocyte lineage after co-culture. The four top images reproduce the more representative phenotypes acquired by AEC (monolayer, circular aggregates, elongated, and tendon-like structures) during the 4 weeks of co-incubation. The bigger images were obtained with the aid of a contrast microscope, while the small ones, inserted in the corner, represent a low magnification recorded under a stereomicroscope. In the lower part of the Table, the incubation intervals (expressed in weeks) required to obtain these different phenotypes are indicated. Scale bar for all images = 50 µm.

**Figure 3:**
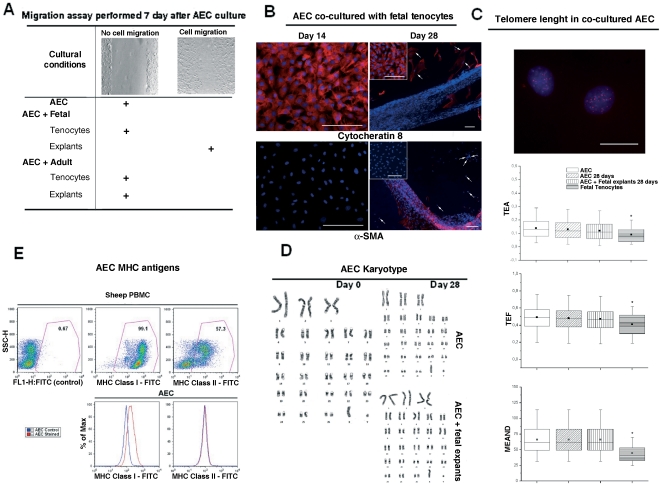
Molecular, genomic, and functional characterization of co-cultured AEC. **A**) The top images are two representative examples of migration testes perfomed in cultured and co-cultured AEC during the first week of incubation. As indicated by the+symbol, only the AEC co-cultured with fetal tendon explants show a migration activity. **B**) Representative images of immunocytochemistry showing the distribution of cytokeratin 8 and α-SMA in AEC co-cultured with fetal primary tenocytes after 14 or 28 days of incubation. All images show cell nuclei in blue (DAPI) and both the proteins in red (Cy3). At day 14, the co-cultured AEC organized in monolayer display high levels of cytokeratin 8, and undetectable levels of α-SMA. A similar molecular phenotype is displayed by monolayered AEC cultured alone (small insets) or co-cultured with fetal tenocytes (arrows in large figures) for 28 days. By contrast, an opposite behaviour is observed in co-cultured AEC that organized cell-aggregates. Scale bar for all images = 100 µm. **C**) Q-FISH detection of telomere length in freshly isolated AEC (AEC), in cultured AEC (AEC 28 days), in AEC co-cultured with fetal explants (AEC+fetal explants 28 days), and fetal tenocytes. The top figure is a representative image showing several hybridized red telomeres (Cy3) within two interphase nuclei stained in blue with DAPI. Scale bar = 15 µm. The three box plots indicate the Telomere area (TEA), the feret maximum (TEF), and the mean densitometric value (MEAND) parameters. The horizontal lines express the 5th, 25th, 50th, 75th, and 95th percentile of the distribution. The box stretches from the 25th to the 75th percentile, and therefore contains the middle half of the scores in the distribution. The median is shown as a line across the box, meanwhile the mean value as a black square within the box. ^*^ indicates data of TEA, TEFmax, and MEAND that resulted significantly different from AEC for *p*<0.01 after One Way ANOVA test followed by *post-hoc* Tukey test. **D**) Three representative normal karyotypes obtained by freshly isolated AEC (day 0), cultured AEC and cells co-cultured in the presence of fetal explants. **E**) Flow cytometry for the major histocompatibily (MHC) class I and II molecules performed on ovine peripheral blood mononuclear cells (PBMC; top image) to test the ovine reactivity of both the antibodies and on freshly isolated AEC (bottom images) to demonstrate the presence of the MHC class I and the absence of MHC class II antigens.

AEC maintained a typical polyhedral shape and did not display any tendency to form three dimensional (3-D) aggregates once confluence was reached (Fig. 2). The expression of cytokeratin 8 persisted, while alpha smooth muscle actin (α-SMA), on the contrary, showed very low levels of expression (Fig. 3).

By contrast, AEC co-cultured with tendon explants or tenocytes assumed different levels of cell organization (Fig. 2).

In detail, during the first 2 weeks of incubation all co-cultured AEC formed a coherent cellular sheet attached on the bottom of the well (Fig. 2). The cell sheets developed quickly several 3-D circular aggregates that appeared at the end of the first week of incubation in AEC co-cultured with fetal tendon explants and later under the other co-cultural conditions. The 3-D circular aggregates grew and progressively elongated, thus detaching from the monolayer substratum but maintaining some peripheral contacts with the well walls. The morphological evolution of the 3-D circular aggregates occurred earlier (14^th^ day) in AEC co-cultured with fetal tenocytes or explants, while, it required 3–4 weeks in AEC exposed to adult derived cells/tissues. Then, the 3-D elongated structures developed more organized tendon–like structures displaying fusiform aligned cells with flat nuclei (Fig. 2, 3). In particular, in AEC co-cultured with fetal explants or fetal tenocytes, a mean of two-three tendon-like structures were observed within each well with a size ranging from 0.4 mm to 2 mm in length. The majority of co-cultured AEC migrated within the 3-D structures and only a very low percentage remained plated on the well bottom (<10%). The tenocytes cultured within the trans-well compartment did not generate organized 3-D tendon-like structures. AEC maintained under different co-culture systems displayed a doubling time ranging from a maximum of 33 hours (adult explants) to a minimum of 23 hours (fetal explants) during the first week of incubation, as summarized in Table 1. The proliferation index recorded in AEC co-cultured during the second week of incubation with fetal explants or fetal tenocytes was slightly higher than that recorded in AEC alone (∼40 and 30%, respectively: *p*>0.05 *vs.* AEC; Table 1), but significantly higher than that of AEC co-cultured with adult derived tendon or tenocytes (∼19 and 12%, respectively; *p*<0.05 *vs.* both fetal co-cultures). The differences amongst the proliferation index persisted (Table 1): the highest proliferation index was recorded in AEC co-cultured with fetal tendon explants (∼40%), while a lower proliferation index was observed in AEC co-cultured with adult explants (∼25%) and fetal tenocytes (∼30%). The lowest proliferation index was observed in AEC exposed to adult tenocytes (∼8%).

### Co-culture induced AEC epithelial-mesenchymal transition

AEC maintained in culture their polyhedral shape, a high incidence of cytokeratin 8 positivity, and did not express α-SMA. The AEC did not display any migratory activity during the first week of incubation (Fig. 3A).

By contrast, the expression of cytokeratin 8 progressively dropped in AEC incubated in co-culture, in parallel with the organization of 3-D cell aggregates. In fact, the AEC that remained plated on the well bottom conserved a high level of cytokeratin 8 positivity, did not express α-SMA, showing the conservation of the original epithelial phenotype (Fig. 3B arrows). By contrast, all the cells that formed the 3-D aggregates down regulated cytokeratin 8 and switched-on α-SMA. This aspect became particular evident in the tendon-like structures, as shown in Fig. 3B. The above epithelial-mesenchymal transition was first observed in AEC exposed to fetal tendon explants (day 7), the only cells that acquired the ability to migrate (Fig. 3A).

The MHC class I and II antigens, telomere length and karyotype were not affected by the cultural and co-cultural conditions. In detail, telomere length was not affected by 28 days of incubation independently from the cultural conditions adopted (Fig. 3C). More in detail, freshly isolated AEC displayed an area (TEA) of 0.14±0.09, a feret maximum (TEF) of 0.49±0.18, and a mean densitometric value (MEAND) of ∼66.48±24.51 that remained stable (TEA 0.13±0.07; TEF max 0.48±0.16; MEAND 66.43±23.84: *p*>0.05 for all the parameters) after 28 days of incubation. Similarly, TEA ranged between a minimum of 0.11±0.06 and a maximum of 0.12±0.09 in AEC co-cultured with fetal and adult tendon explants, respectively; the TEF maximum ranging between a minimum of 0.47±0.14 and 0.48±0.12 in AEC co-cultured with fetal tendon explants and adult tenocytes, respectively; MEAND ranged between a minimum of 64.89±21.89 and a maximum of 65.28±20.56 in AEC co-cultured with fetal and adult tenocytes, respectively. The telomere parameters recorded in the different groups of co-cultured AEC were similar to those recorded in freshly isolated AEC (*p*>0.05 for all parameters) and cultured AEC (*p*>0.05), but significantly greater than those recorded in fetal tenocytes (TEA 0.08±0.05, TEF max 0.40±0.13, MEAND 44.01±13.24; *p*<0.05 for all parameters; Fig. 3C).

The structures and the modal number of chromosomes analyzed in undifferentiated (time 0 and 28 days; Fig. 3) and differentiated AEC indicated a constant chromosomal number (n = 54; Fig. 3D) and confirmed the absence of gross chromosomal instability during the incubation.

All the tested AEC, independently from the cultural conditions adopted, maintained a similar and stable expression profiles for MHC class I and II antigens. In particular, freshly isolated (Fig. 3E), cultured and co-cultured AEC displayed a low expression of MHC class I molecule (MFI ratio ranging between 2.5 and 4), and the absence of MHC class II (HLA-DR) antigens (MFI ratio always >1).

### Fetal explants and tenocytes triggered AEC tenogenic differentiation

Immunohistochemistry revealed that all the co-cultured AEC progressively increased their intracytoplasmic content of COL1 protein.

**Figure 4:**
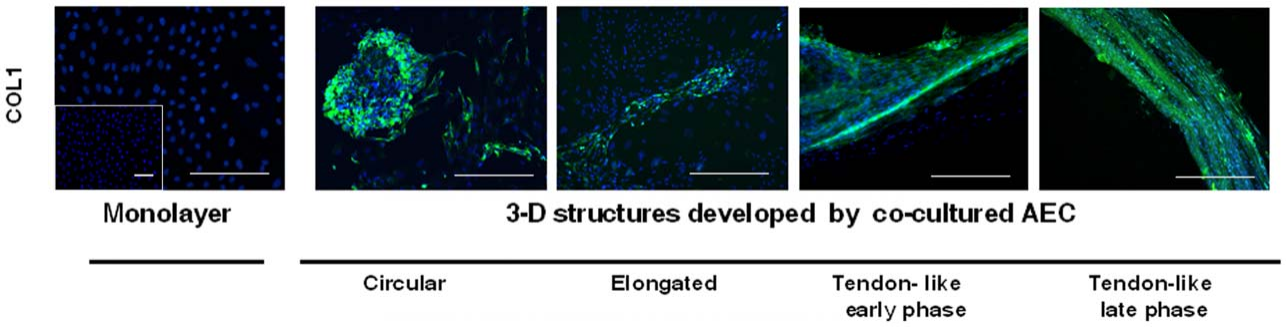
COL1 distribution in AEC aggregates developed in co-culture. The images exemplify the COL1 protein distribution recorded by immunohistochemistry in the more representative typologies of cell aggregates obtained during AEC co-cultures. The images show in blue the nuclei counterstained with DAPI and in green the COL1 protein (Alexa Fluor 488). The COL1 protein is undetectable when the AEC are organized in monolayer independently from the cultural conditions adopted (co-cultured AEC, monolayer; AEC cultured alone, small inset). COL1 starts to appear in AEC forming cell aggregates and reaches its highest and widespread distribution within the tendon-like structures. In the early phase, COL1 is localized within the fusiform cells that start to be oriented along the longitudinal axis of the tendon-like structures. Later, the protein was either localized into the AEC or deposited within the extracellular matrix (tendon-like: late phase). Scale bar for all images = 100 µm.

Large amounts of COL1 appeared in AEC forming the 3-D aggregates (Fig. 4). On the contrary, the protein was not detectable for 28 days either in AEC cultured alone or in AEC forming the monolayer (Fig. 4).

**Figure 5:**
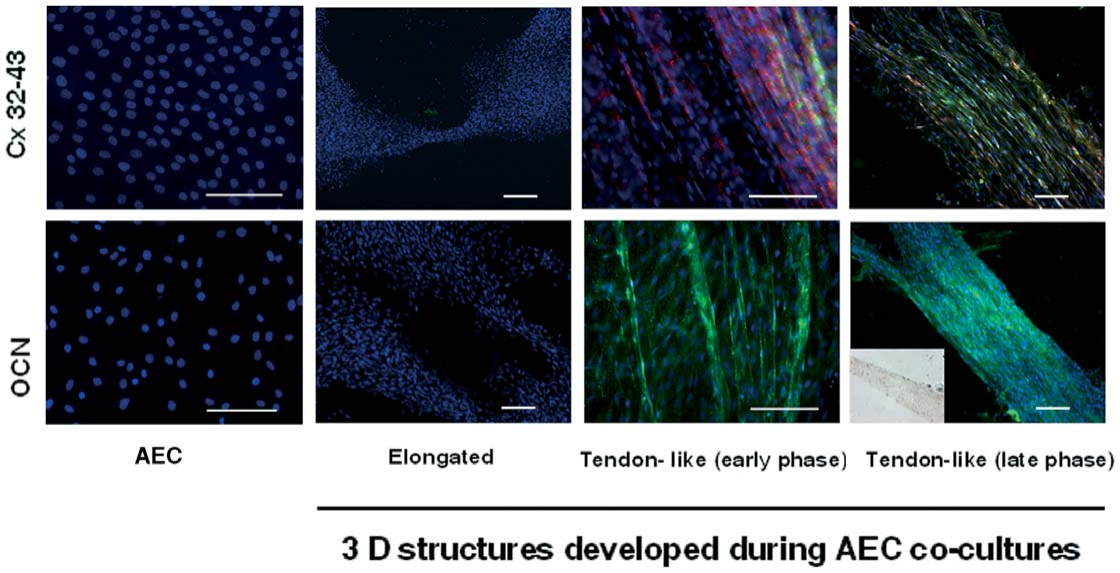
OCN, Cx32 and Cx43 proteins distribution in AEC co-cultured with fetal tenocytes or tendon explants. Representative images showing osteocalcin (OCN: lower panel) and connexins (Cx32 or Cx43: upper panel) immunolocalization in AEC cultured alone (AEC) and in AEC co-cultured fetal derived cell/tissues that developed 3D structures. The pictures show the cell nuclei in blue (DAPI), OCN or Cx32 proteins in green (Alexa Fluor 488) and Cx43 in red (Cy3). The co-expression of Cx proteins on AEC cell aggregates were analyzed with a double immunostaining. OCN and both the Cx proteins were undetectable in AEC cultured alone (AEC) and in AEC organized within elongated aggregates. By contrast, AEC that differentiated 3-D tendon-like structures co-expressed Cx32 and Cx43. The Cx43 protein shows higher levels in the early tendon-like structures with a clear membrane localization, while Cx32, more abundant in late structures, is localized either on the membrane or into the cytoplasm. OCN appears as a cytoplasmatic protein within the fusiform shaped cells forming the tendon-like structures (early phase). Its intracellular levels progressively increased during the process of *in vitro* tendon differentiation (late phase). As indicated in the corner box showing a representative image of an ALP assay, tendon-like structures did not display any osteogenic foci. Scale bar for all images = 50 µm.

In detail, the highest levels of COL1 were recorded within the more organized tendon-like structures developed *in vitro* by AEC exposed to fetal tendon explants or fetal tenocytes. In these structures the protein was first observed within the fusiform cells (early phase) paralleled oriented and, then, it appeared in the extracellular spaces (late phase; Fig. 4). The tendon-like structures were the only 3-D structures that expressed OCN, connexin (Cx) 32 and 43 (Fig. 5). In fact, while COL1 positive cells were present in all the 3-D structure (Fig. 4), OCN and Cx proteins were observed only in tendon-like structures (Fig. 5). The OCN was clearly localized into the cytoplasm of the fusiform shaped cells forming the 3-D tendon-like structures where no ALP positivity was observed, thus confirming the absence of any osteogenic *in vitro* differentiation (inset in Fig. 5). Moreover, the *in vitro* differentiated AEC forming 3-D tendon-like structures co-expressed Cx 32 and 43. During the early phase of 3-D structure formation several cells displayed Cx 43 punctuate foci on the membranes. Later, the tendon-like structures showed high levels of both proteins that connected the fusiform cells to each other, thus forming a syncytial functional network. The fusiform cells produced large amounts of Cx 43 that resulted localized either on the membrane or into the cytoplasm, suggesting a protein transfer from the cytoplasm to the membrane (Fig. 5).

### Co-cultures affected expression profile of tendon/ligament related genes

**Figure 6:**
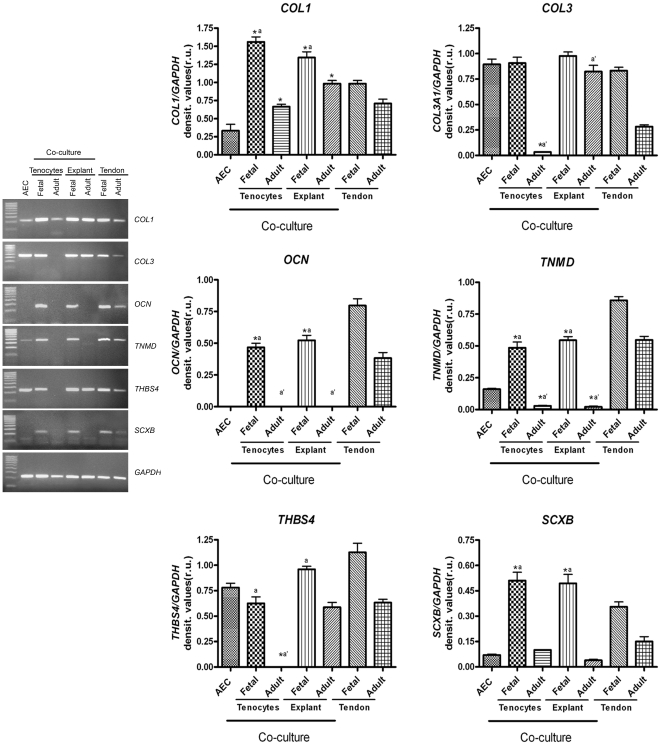
Expression profile of tendon/ligament-related genes in co-cultured AEC. The mRNA content of *SCXB*, *COL1*, *COL3*, *TNMD*, *THSB4*, *OCN* was analyzed in cultured AEC or in AEC co-cultured with fetal or adult tenocytes/tendons explants by using RT-PCR. The semi quantitative analyses of mRNA levels were normalized for *GAPDH* gene and expressed as mean of 3 different replicates ± SD. The data were compared by One Way ANOVA test followed by *post-hoc* Tukey test. ^*^ Values significantly different from AEC group for *p*<0.05; **^a^** values significantly different from fetal tendon group for *p*<0.05; **^a′^** values significantly different from adult tendon group for *p*<0.05.

As summarized in Fig. 6, gene expression profiles performed after 28 days of incubation confirmed that fetal co-cultures (tendon explants or tenocytes) were more effective in promoting tenogenic *in vitro* differentiation.

*SCXB* and *TNMD*, two specific tendon/ligament related genes, highly expressed in native ovine adult or fetal tendons, were significantly up regulated in AEC co-cultured with fetal explants or fetal tenocytes (*p*<0.05 *vs.* AEC alone). By contrast, *SCXB* mRNA content was low in AEC co-cultured with the adult tissue or primary adult tenocytes (*p*>0.05 for both *vs.* AEC) where, in parallel, *TNMD* mRNA levels significantly decreased (*p*<0.05 *vs.* AEC). Analogously, *OCN* was switched-on exclusively in AEC exposed to fetal tissues/cells even if their mRNA content was lower than that recorded in fetal and adult tendons (*p*<0.05 for both *vs.* fetal tendon). In parallel, AEC incubated under fetal co-cultures up regulated *COL1* that reached significantly higher values than those recorded in cultured AEC (*p*<0.05 for both) and observed in native tissues (*p*<0.05; Fig. 6). By contrast, fetal co-cultures did not affect *COL3* and *THBS4* mRNA content.

Adult co-cultures stimulated different expression profiles (Fig. 6). In detail, the *COL3* and *THBS4* mRNA contents remained stable in AEC exposed to adult tendon explants, while they dropped in cells co-cultured with adult tenocytes. By contrast, adult tenocytes or explants significantly increased the mRNA levels of *COL1* and decreased *TNMD* mRNA content, respectively. Moreover, both the adult co-cultures were unable to up regulate the expression of *OCN* and *SCXB*.

## Discussion

The present work demonstrates, for the first time, that ovine AEC can be differentiated into tenocytes by using an appropriate co-cultural microenvironment. The AEC may, thus, represent a promising source of tenocyte/tenocyte progenitor cells which together with MSCs and embryonic stem cells (ESC) can be used to develop cells for transplantation or for the production of engineered tendons [8]. This aspect may have crucial clinical relevance because the extremely low availability of autologous tenocytes or progenitor cells has so far strongly limited the development of a cell-based therapy for tendinopathy [3], [4]. Amniotic membranes, that in human could be collected by the term placenta as a discarded tissue with few ethical issues [22]–[24], may provide a large amount of AEC. Amniotic derived cells for veterinary medicine could be advantageously obtained directly from the placenta of slaughtered pregnant animals.

More in detail, the present paper showed that ovine AEC can stepwise differentiate *in vitro* into tenocytes through an epithelial-mesenchymal transition stage. The process is more consistent in co-culture with fetal derived cells/tendon explants that provides a powerful and low cost strategy for improving tenogenic *in vitro* differentiation.

Taking into account that the regenerative properties of a tissue is strictly age-related [25]–[28], we have compared the inductive properties of fetal *vs.* adult tendon samples in co-culture. Several authors demonstrated, in fact, that the complex interaction between extrinsic and cell-intrinsic influences limit tissue regeneration during the transition from fetus to adult life. Extrinsic factors, such as changes in the precursor cell niche, local or systemic signals, exert a primary role in concert with cell intrinsic pathways [29], [30]. These aspects are particularly relevant when adult and fetal tissue regenerative properties are compared. In contrast to adult tissue repair, extensive experimental evidences showed that fetal tissues in early and mid-gestational stage, like that used in the present work, respond to injuries in a highly efficient manner. In general, fetal wound healing occurs at a faster rate and without any scar formation in different animal models [31], [32]. Recent investigations demonstrated that these high regenerative properties are intrinsic to fetal tendon itself [33], [34]. Cells isolated from fetal ligament exhibit enhanced cellular migration and collagen production [28] in comparison to cells from adult tissue. In addition, sheep fetal tendon transplanted into an adult environment responds to injuries in an intrinsic manner of the tissue itself by displaying a rapid morphological regeneration, and by recovering normal mechanical properties [33]. Although the mechanisms involved are still unknown, these data strongly indicate that the intrinsic regenerative capability of the fetal tendon may be dependent on specific intracellular pathways and/or the local secretion of peculiar paracrine factors that could explain the maintenance of the regenerative properties of the transplanted fetal tendon, and the fibrotic evolution in an adult injured tendon. The co-culture systems used did not require any cell-cell interaction, but simply operated under the stimulatory influences of paracrine humoral factors released by the different inductive samples. Under these co-culture systems we were able to discriminate between the tenogenic inductive properties exerted by fetal *vs.* adult and cells *vs.* tissue explants.

Fetal samples were able to direct AEC into a fully differentiated tenogenic phenotype.

Firstly, fetal co-cultures increased the AEC proliferative activity and induced a prompt epithelial-mesenchymal transition as documented by cytokeratin 8 and α-SMA expression, and by the acquisition of a clear migration ability. Epithelial-mesenchymal transition occurred during the first two weeks of incubation and was accelerated by fetal tendon explants. The higher stimulatory inductive properties of fetal samples (explants and tenocytes) were then confirmed by the analysis of cell morphology, of the 3-D aggregate structures, and by *SCXB*, matrix (*TNMD*, *COL1* and *3*, *THBS4*), and mesenchymal (*OCN*) gene expression. The combination of the above parameters was adopted as the only way to interpreter the degree of *in vitro* tenogenic differentiation in the absence of any specific tendon tissue markers [8], and of ultrastructural analysis showing the collagen fibrils in tendon-like structures [35]. The study of gene expression, however, well describes the tenogenic evolution of the co-cultured AEC showing that only after co-culture with fetal samples, AEC assumed an expression profile similar to that of native ovine adult and fetal tendons.

Amongst the considered different genes, the expression of *SCXB* promoted by fetal samples appeared the more remarkable event, considering that SCXB is the best characterized tendon neo-formation molecular marker [36], [37]. SCXB, in fact, is a member of the basis-helix-loop-helix (bHLH) super-family of transcription factors involved in developmental processes such as tendon formation and tendon muscle attachment during fetal life [38]. Additional evidences suggested a positive role of *SCXB* also in adult tendon homeostasis as co-activator of other tendon correlated genes as *COL1* [39] and *TNMD* [40]. Analogously, the induction of *SCXB* gene expression in AEC exposed to fetal paracrine factors was, in parallel, accompanied by the switching on of *TNMD*, and *OCN* as well as by the up regulation of *COL1*.

Similarly, the global gene profile expression clearly showed the different tenogenic potential of adult and tendon explants and adult primary tenocytes. Adult primary tenocytes, that represent the more used source of cells for the co-cultural systems [18], [19], showed, actually, the lowest tenogenic differentiative influence on ovine AEC. Adult tenocytes, in fact, displayed a lower ability than fetal tenocytes to stimulate AEC proliferation, and to induce mesenchymal transition. In addition, in the presence of adult tenocytes AEC down-regulated the expression of matrix-related genes as *COL3*, *TNMD*, and switched off the *THSB4*.

Co-cultured ovine AEC were able to form coherent cellular sheets and yield tendon-like structures in the absence of a 3-D culture system [19], scaffold support [41], [42], and any mechanical stimuli [8]. The tendon-like structures reached high levels of organization in terms of morphological and molecular phenotypes, in particular, in the presence of fetal cells/tissues co-cultures. In fact, the *in vitro* tendons obtained with fetal soluble factors contained layered fusiform cells that expressed high intracellular levels of COL1 and OCN proteins. OCN is considered predominantly a marker associated to osteogenesis, but other reports indicated that this protein is expressed in tendon and tendon cell lineages [15]. In the present work, OCN was chosen to confirm the new protein profile induced in AEC by co-culture since anti-sheep SCXB and TNMD antibodies specific for immunohistochemestry are not commercially available. In order to exclude a possible correlation between OCN and osteogenic foci in the *in vitro* produced tendon, however, the ALP negativity was, in parallel, documented.

The tendon-like structures developed in co-cultures with fetal derived cells/tendon explants, in addition, were the only 3-D structures displaying COL1 proteins deposited in the extracellular matrix.

The higher level of organization of these tendon-like structures was, finally, suggested by the expression of both Cx proteins forming the gap junction communication system typical of fetal and adult tendons [43]. The gap junctions that link tenocytes within the native tissue represent a functional network allowing mechanical coordination and strain-induced collagen synthesis [44]–[47]. A 3-D network of gap junctions appeared in the tendon-like structures after 3–4 weeks of co-incubation with fetal derivates, enabling them to respond potentially better to mechanical stimuli that could be exploited to further improve their organization [8].

In conclusion, a highly efficient co-culture system capable to program AEC through tenogenic soluble factors has been identified.

The co-cultural system with fetal cell/tendon derivates guaranteed the formation of tendon-like structures for biological study and cell-therapy application when applied to AEC or other MSCs. The co-culture system, in addition, appears able to conserve the AEC characteristics since it did not affect telomere length, karyotype, and the expression of histocompatibility antigens of class I and II.

In particular, the HLA expression of ovine AEC reproduced what it has been demonstrated in human AEC, thus suggesting that these cells may be applicable in clinical transplantation settings under auto/allo/xenogeneic conditions [48]–[52]. The autologous use of amniotic-derived cells has been proposed for the uterus cell transplantation aimed to treat congenital disorders [53], or for cell-based therapies in adult life after AEC bio-banking [22]–[24]. The allo/xenogeneic use has been also hypothesized as a consequence of the AEC immunomodulatory [52], [54]–[56], and low tumorigenic properties [22]–[24]. However, independently of the therapeutic use of amniotic-derived cells, accurate long term preclinical/clinical studies to evaluate AEC safety and stability in host tissues are still required. In this context, one future target of our group will be to compare the allogeneic stability and regenerative properties of undifferentiated *vs.* differentiated AEC when injected into an experimental induced tendon defect [57], [58] performed on a medium sized mammal, the sheep, selected for its high translational value. Before the allotransplantation of the differentiated AEC into immunocompetent animals, their immunogenicity has to be tested trying to adapt the methods previously described in other animal models [52], [54]–[56].

Albeit, the study of the tenogenic regenerative properties of AEC is of high value, this is not the aim of the present work that it has been addressed to identify a low cost and efficient *in vitro* cultural protocol able to stimulate tenogenic differentiation that here it has been applied to ovine AEC.

The major limitation to translate this procedure in medicine may be represented by the low availability of tendon/tenocytes and, in particular, tendon/tenocytes of fetal origin, the co-culture performed with sheep tenocyte/tendons may represent a cellular culture system on which either identify the molecules involved into the process of tenogenic *in vitro* differentiation or to produce high amounts of conditioned media to use for human AEC/MSCs differentiation.

## Materials and Methods

### Ethics statement

All cells and tissues were collected from slaughtered animals, and this did not require an ethic statement.

### AEC and tendon explants/tenocytes isolation and culture

In the present research, the co-culture system was used in order to compare the inductive tenogenic potential of adult and fetal tendon explants, or adult and fetal primary tenocytes on freshly isolated ovine AEC. To this aim, adult calcaneal tendons and fetal tissues (amniotic membrane and calcaneal tendons) were obtained by slaughtered sheep. Adult tendons were collected by adult female sheep, 2–3 years old, of Appenninica breed. The amniotic membranes and fetal tendons were isolated from fetuses of 25–35 cm of length, at approximately 2–3 months of pregnancy.

Once opened the uterus wall, the AEC were collected from pieces of approximately 3–5 cm of the amniotic membranes that were mechanically peeled off. Membrane pieces were washed in Phosphate Buffered Saline (PBS; Sigma Chemical Co. St. Louis, MO), and incubated in 0.25% Trypsin/EDTA 200 mg /L at 37.5°C for 20 minutes under gentle agitation. Then, cell suspension was collected, filtered through a 40 µm cell filter and poured into a 50 ml falcon tube containing FCS at a final concentration of 10% to inactivate trypsin. Each falcon tube was centrifuged, and the pelleted vital cells were counted after trypan-blue staining by using a haemocytometer chamber. Isolated cells were, then, used for flow cytometry analysis and for co-cultural experiments.

Calcaneal tendon explants were isolated from the forefeet and deprived of the peritendineum under sterile conditions. Small pieces of fresh tendon isolated from the middle portion of the structures (about 1 mm^3^ in size) were further mechanically disaggregated under a stereomicroscope with the aid of fine watchmaker forceps in order to maximize the interface between tissue and medium. Tendon explants were immediately placed on the membranes of trans-wells (membrane porosity 0.4 µm) of a 12 well chamber (Costar, NY, USA).
Primary adult and fetal tenocytes were isolated after *in vitro* incubation of calcaneal tendon explants. The culture were performed in petri dishes of 25 mm containing 2 ml of α-MEM supplemented with 10% FCS and 1 mL/100 mL L-glutamine and antibiotics/antimycotic solution (penicillin G sodium 100 U/ml, streptomycin 100 µg/ml, amphotericin B 0.25 µg/ml; Gibco, Invitrogen, Carlsbad, CA, USA) at 38°C in 5% CO_2_ and air.

Tenocytes were, then, isolated by using 0.25% Trypsin/EDTA solution when the cells migrated out of tendon pieces and reached 60–80% of confluence (∼4 days for fetal tendon explants and ∼9 days for adult tendons). Collected tenocytes were immediately used for the co-culture systems in order to avoid their rapid phenotype drift with further *in vitro* passages [59].

### Co-culture systems

The co-culture system was performed as described by Luo et al. [18], with minor modifications. In detail, transwell chambers (pore size 0,4 µm; Costar, NY, USA) containing primary tenocytes (1×10^4^ cells/trans-well) or tendon explants (two-three explants of 1 mm^3^ in size/trans-well) were inserted into the wells plates. The AEC were plated onto the well plates at 1×10^4^ cells/well. Each trial was performed by simultaneously comparing the four different conditions of co-culture (fetal and adult tendon explants or primary tenocytes) each carried out at least in triplicate. The incubation was performed in α-MEM supplemented with 10% FCS in 5% CO_2_ and air at 38°C for 28 days. Half of the medium was changed every 3 days during the first week of culture, and then, every 1–2 days. The AEC *in vitro* differentiation was monitored as described below.

### AEC characterization

Freshly isolated AEC were immediately screened by flow cytometry for the surface molecules CD14, CD29, CD31, CD45 CD49, CD58, CD117 and CD166 and for intracellular stem cell markers (TERT, OCT4, SOX2, NANOG), as detailed below. The primary antibodies used for the analysis were purchased as indicated in Table 2. The un-conjugated primary antibodies were FITC marked by using Zenon Antibody Labelling Kit (Gibco, Invitrogen, Carlsbad, CA, USA), following the manufacturer's instructions. Staining for flow cytometry were performed on 5×10^5^ cells/sample by incubating them with 100 µl of 20 mM ethylenediaminetetraacetic acid (EDTA) at 37°C for 10 minutes. Cells were, then, washed in 3 ml of washing buffer (PBS, 0.1% sodium azide and 0.5% Bovine Serum Albumine, BSA), and centrifuged (4°C, 400×g, 8 minutes). For surface antigens staining, cell samples were suspended in 100 µl washing buffer containing the appropriate amount of surface antibody; samples were incubated for 30 minutes at 4°C in the dark. Tubes were washed (3 ml of washing buffer), centrifuged (4°C, 400×g, 8 minutes) and cells were suspended with 1 ml 0.5% paraformaldehyde, incubated for 5 minutes at room temperature (RT), washed, centrifuged (4°C, 400×g, 8 minutes) and stored at 4°C in the dark until the acquisition. For intracellular antigens staining, cells were suspended in 1 ml of FACS Lysing solution (BD), vortexed and incubated at RT in the dark for 10 minutes. Samples were, then, centrifuged (4°C, 400×g, 8 minutes); 1 ml of Perm 2 (BD) was added to each tube, and cells were incubated at RT in the dark for 10 minutes. Samples were washed (3 ml of washing buffer) and centrifuged (4°C, 400×g, 8 minutes). Cells were suspended in 100 µl of washing buffer containing the appropriate amount of intracellular antibody and incubated for 30 minutes at 4°C in the dark. Tubes were centrifuged (4°C, 400×g, 8 minutes), and cells suspended with 1 ml 0.5% paraformaldehyde, incubated for 5 minutes at RT, washed, centrifuged (4°C, 400×g, 8 minutes), and stored at 4°C in the dark until the acquisition. Finally, cells were analysed on a FACSCalibur flow cytometer (BD), using CellQuest™ software (BD). Flow Cytometer Measurement was carried out by using as quality control Rainbow Calibration Particles (6 peaks) and CaliBRITE beads (both from BD Biosciences). Debris were excluded from the analysis by gating on morphological parameters (lymphocyte gate); 20.000 non-debris events in the morphological gate were recorded for each sample. All antibodies were titrated under assay conditions and optimal photomultiplier (PMT) gains were established for each channel [60]. Data were analysed using FlowJo™ software (TreeStar, Ashland, OR). Mean Fluorescence Intensity Ratio (MFI Ratio) was calculated dividing the MFI of positive events by the MFI of negative events [61].

**Table 2:** Details of primary antibodies used in flow cytometry analysis.

Antigen	Conjugated-fluorescent probe	Company details	
***Hemopoietic markers***			
CD14	FITC	LifeSpan Bioscences	Seattle, WA, USA
CD58	FITC	LifeSpan Bioscences	Seattle, WA, USA
CD31	FITC	AbD Serotec	Oxford, UK
CD45	FITC	AbD Serotec	Oxford, UK
***Adhesion molecules***			
CD29		VMRD	Pullman, WA, USA
CD49f		Beckman Coulter	Fullerton, CA, USA);
CD166	FITC	Ancell	MN, USA
***MHC antigens***			
Class I		Novus Biologicals	Cambridge UK
Class II HLA-DR		Abcam	Cambridge UK
***Stemness markers***			
CD117		Abcam	Cambridge, UK
SOX2		Abcam	Cambridge, UK
OCT4	PE	Becton Dickinson	BD, San Jose, CA
TERT		Calbiochem	Gibbstown, NJ
NANOG		Chemicon Int.	Billerica, MA

Finally, the plasticity and differentiation potential of ovine AEC was tested *in vitro* by adopting validated methods to differentiate amniotic derived cells in endoderm (liver) [62], mesoderm (bone) [63], and ectoderm (neural cells) [64] cell lineages. In detail, for hepatic differentiation, freshly isolated AEC cells were allowed to proliferate for 2 days on well plates coated with Matrigel (BD Bioscience) in α-MEM supplemented with 10% FCS and 10 ng/ml epidermal growth factor (EGF; Sigma Chemical Co. St. Louis, MO). On day 2, the medium was substituted with Iscove's modified Dulbecco's medium (Lonza, Walkersville, MD) enriched with 10% FCS, 10 ng/ml EGF and 10 ng/ml of fibroblast growth factor (FGF 2; R&D Systems Inc., Minneapolis) for 48 hours, and then, supplemented with 20 ng/ml of hepatic growth factor (HGF; Gibco, Invitrogen, Carlsbad, CA, USA), 10^−6^ M dexamethasone and 1% insulin/transferrin/selenium (Sigma Chemical Co. St. Louis, MO), and the incubation was maintained for further 5 days. Thereafter, the treatment was continued for an additional week, with one exception: FGF 2 was replaced with 20 ng/ml Oncostatin-M (Peprotec, Rocky Hill, NJ). Hepatic differentiation was evaluated by the immunocytochemical expression of albumin (Table 3). In order to evaluate the species-specificity of the used antibody, primary mouse and ovine hepatocytes were used as positive controls. Osteogenic differentiation was induced in a standard osteogenic medium (α-MEM supplemented with 50 µM ascorbic acid, 10 mM β-glicerol phosphate, 0.2 µM dexamethasone and 10% FCS) for 15 days, and assessed by the extracellular matrix mineralization with Alizarin Red staining. Neural differentiation was induced in standard medium supplemented with 5×10^−5^ M all-trans retinoic acid (Sigma Chemical Co. St. Louis, MO) and 10 ng/ml FGF (R&D Systems Inc, Minneapolis, MN), for 10 days before assessing the immunocytochemical expression of nestin (Table 3). In order to evaluate the specie-specificity of the used antibody, human Glioma U87 cell line and cryo-sectioned ovine myo-tendinous junctions were used as positive controls.

**Table 3:** Details of primary and secondary antibodies used for immunohistochemistry.

Primary Abs (Company details)	Primary Ab dilutions	Secondary Abs (Company details)	Secondary Ab dilutions
**Ki-67** (Dako Cytomation, Denmark)	1∶50	Anti-Mouse Alexa Fluor 488 (Invitrogen Ltd, Paisley, UK)	1∶100
**COL1** (Chemicon Int. Billrerica, MA)	1∶100	Anti-Mouse FITC (Sigma-Aldrich, Missouri, USA)	1∶500
**Cytokeratin 8** (Abcam, Cambridge, UK)	1∶200	Anti-Mouse Cy3 (Sigma-Aldrich, Missouri, USA)	1∶500
**α-SMA** (Abcam, Cambridge, UK)	1∶500	Anti-Mouse FITC (Sigma-Aldrich, Missouri, USA)	1∶100
**Albumin (V-14)** (Santa Cruz Biotechnology, California, USA)	1∶100	Anti-Goat Alexa Fluor 488 (Invitrogen Ltd, Paisley, UK)	1∶250
**Nestin, clone 10C2** (Millipore, Billerica, MA)	1∶250	Anti-Mouse Cy3 (Sigma-Aldrich, Missouri, USA	1∶500
**Cx43** (Chemicon Int. Billrerica, MA)	1∶200	Anti-Mouse Cy3 (Sigma-Aldrich, Missouri, USA)	1∶500
**Cx32** (Chemicon Int. Billrerica, MA)	1∶200	Anti-Mouse Alexa Fluor 488 (Invitrogen Ltd, Paisley, UK)	1∶750
**Osteocalcin** (Abcam, Cambridge, UK)	1∶50	Anti-Mouse Alexa Fluor 488 (Invitrogen Ltd, Paisley, UK)	1∶400

Primary and secondary antibodies (Abs) are diluted in PBS supplemented with 1% BSA.

### Morphological and functional AEC evaluation after co-culture

AEC morphology during the *in vitro* co-culture was followed every 7 days under an inverted microscope. At each interval points, AEC co-cultures were stopped and used for the following analyses.

In detail, the proliferative activity was analyzed until AEC reached the monolayer by calculating their doubling time. When the AEC started to generate in culture three dimensional structures, the mitotic activity of the cells was deduced by the proliferation index. This parameter was recorded by using the molecular marker Ki-67, a nuclear and nucleolar protein strictly associated with cell proliferation [65], [66]. The proliferation index was calculated by counting the number of Ki-67 positive cells/total cells counterstained with DAPI. To this aim, for each experimental group three replicates were considered obtained from three different experiments. Two trained observers blinded to the experiment counted at least 200 cells for each replicate.

In addition, an *in vitro* migration assay was performed according to Stalling [28] in order to monitor the epithelial-mesenchymal transition. Before AEC aggregation occurred, co-cultured cells were trypsinized and put over a Nunc cover slide chamber (4 cm^2^) containing in the middle a 1-mm wide silicone rubber strip. A total of 2×10^6^ cell/ml cell was seeded. After 1 hour, when the cells started to adhere, each well was covered with α-MEM containing mitomycin to stop cell proliferation. Twenty four hours later, silicone strips were carefully removed. Wells were rinsed gently in order to remove cellular debris, and cell recording started (Axiovert 200 & Axiovision; Carl Zeiss, Thornwood, NY). The analyses were performed by capturing the field images after 16 hours (Axiovision and KS300; Carl Zeiss) to track migration. The width of the cell-free zone was photographed and compared at time 0 and after 16 hours. The migration assays were performed on six different replicates/co-cultural groups.

The *in vitro* stability of the co-cultured AEC was evaluated by comparing telomere sizes, the karyotype, and the expression of MHC antigen Class I and II before and at the end of the incubation interval.

Telomere size detection was performed by using the Quantitative Fluorescence In Situ Hybridisation (Q-FISH) [67]. In detail, confluent cultured and co-cultured AEC were transferred in dishes and incubated a 37°C for 24–48 hours in Chang Medium (Irvine Scientific). Cells were treated with a pre-warmed (37°C) hypotonic solution (0.06 M KCl) before fixing them in 3∶1 methanol∶ acetic acid (Carnoy's fixative). The air dried cover slips were then applied to the slides with a drop of EN-Aquivitrex Erba (Carlo Erba, Italy). Slides were then placed in 2× SSC at 45°C for 5 minutes, digested with pre-warmed (37°C) pepsin solution (1 mg/ml; Sigma) at RT for 7 minutes, thoroughly rinsed with PBS, and fixed in 4% paraformaldehyde/PBS for 3 minutes at RT before dehydration in ethanol series (70%, 95%, 100% at 2 minutes each). Interphase nuclei were denatured with 70% formamide (Carlo Erba) in 2× SSC, pH 7.0 at 75.5°C for 5 min., followed immediately by dehydration in ice-cold ethanol (70%, 95%, 100% at 2 minutes each). A denatured all-human telomeric DNA probe, since eukaryotic chromosomes, as well as the sheep [68], contain conserved non-coding sequences of DNA repeats (TTAGGG)_n_, was added to each slide (Qbiogene – Resnova). The incubation with telomeric DNA probe was performed overnight at 37°C in a humidified chamber, and post-hybridized with 50% formamide (Carlo Erba) in 2× SSC at 37°C for 10 minutes. The hybridized signals were then detected by using a commercial kit (Cy3 avidin detection kit; Oncor Inc.) according to the manufacturer's instructions. DAPI/Antifade mounting medium (Vectashield, Vector Laboraties, Inc. Burlingame, CA) was used for chromatin counterstaining. All slides were analyzed using an Axioskop 2 Plus incident-light fluorescence microscope (Zeiss) equipped with a PL-Neofluorar ×100 oil immersion objective (NA 1.30) and a ×10 ocular to provide images with a spatial resolution of 0.25 µm, a HBO 100 W mercury lamp, a Cy3 filter (excitation: BP546/12; emission: BP575–640; no. 20, Zeiss), and a DAPI filter (excitation: BP 365/12; emission: LP 397; no. 01, Zeiss). The microscope was equipped with a cooled color CCD camera (Axiovision Cam, Zeiss) with a resolution of 1300×1030 pixels, configured for fluorescence microscopy, and interfaced to an image acquisition dedicated software (Axiovision, Zeiss). Digital image analysis of telomere length was performed according to Russo et al. [69]. Since in interphase nuclei telomeres are distributed throughout the nucleus, the focus of the microscope was adjusted so that most telomeres were in focus. For quantitative purposes digital images were captured from at least 100 interphase nuclei in each specimen immediately after hybridization. At the beginning of an imaging session, optimum exposure times were determined and held constant thereafter. In all cases, it was confirmed that the telomeric signals were within the linear response range of the CCD camera using, according to Meeker et al. [70], standard fluorescent microbeads (InSpeck microspheres; Molecular Probes, Inc.). In brief, quantification of the digitized fluorescent telomere signals was accomplished by using a semi-automated algorithm written with the image analysis software package KS300 (Zeiss), as described in Russo et al. [69]. Parameters to be measured included mean densitometric value, area and feret maximum, which corresponds to the value of the major diagonal connecting the two farthest points at the periphery of the object. Results were then recorded for statistical evaluation.

Karyotype evaluation was performed in AEC before and after the different cultural conditions. Cells were harvested from flask after trypsin digestion, transferred in amniodish and then incubated at 37°C for 24–48 hours in Chang medium (Irvine scientific). Metaphase cells were arrested with 0.1 µg/ml colchicine for 2 hours at 37°C. Then, treated with hypotonic solution (0.06 M KCl) and incubated for 20 minutes at 37°C. Cells were fixed in methanol/glacial acetic acid (3∶1) for 10 minutes. Metaphase chromosomes were subjected to GTG-banding and Giemsa staining. A median of 30–40 metaphases were examined for each sample at approximately the 350–450 band level, using a Nikon Eclipse 80i microscope and analyzed by Genikon software (Nikon). A normal ovine karyotype consists in 27 pairs (2n = 54) of chromosomes. A chromosomal aberration was defined as clonal when at least two metaphases showed the same abnormality.

Finally the major histocompatibility antigens of class I and II (HLA-DR) were evaluated before and after co-culture by using flow cytometry as discussed above by using the primary antibodies described in Table 2.

### Immunohistochemistry

In addition, the AEC incubated under different co-cultural systems were, then, tested in order to monitor with immunohistochemistry:

Cytokeratin 8 and α-SMA to test the transition from epithelial to mesenchymal lineage,
OCN, a generic mesenchymal derived tissue marker molecule,
COL1, an abundant tendon matrix component,
Cx 32 and Cx 43, two tendon gap junction proteins.

In detail, immunohistochemistry was carried out on cultured and co-cultured AEC for 7, 14 or 28 days. For the single immunostainings, cells contained in each well were fixed for 10 minutes with 4% paraformaldehyde/PBS. After washing with PBS, cells were permeabilized with 0.1% Triton X-100/PBS for 10 minutes at RT. The AEC were blocked by incubating at RT in PBS/1% BSA for 1 hour. Cells were, then, incubated with the primary antibody (Ab) overnight at RT, and then, exposed to the secondary Ab for 40 minutes at RT. Experiments with the omission of primary Abs were used as negative controls. Cell nuclei were identified with DAPI counterstaining.

Double immunocytochemistry for Cx32 and Cx43: after washing with PBS, cells were blocked at RT in PBS/1% BSA for 1 hour. Samples were then incubated with a mouse monoclonal anti-Cx32 Ab overnight at 4°C. Next, the cells were washed in PBS, and incubated with an anti-mouse Alexa Fluor488 for 60 minutes, and washed again in PBS. After this first step, a post-fixation with 4% paraformaldehyde/PBS for 10 minutes at 4°C was performed. Then, after thoroughly washes, the samples were incubated with a mouse monoclonal anti-Cx43 Ab overnight at RT and revealed with a Anti-Mouse Cy3. All details on the primary and secondary Abs are shown in Table 2.

### Total RNA isolation and RT-PCR

RT-PCR analyses were performed on ovine native tendons (adult and fetal), and on cultured and co-cultured AEC after 28 days of incubation in order to compare the expression of specific genes, summarized in Table 4.

**Table 4:** Primer sequences used in RT-PCR analyses.

Gene	Accession No.	Primer sequences	Product size(bp)	PCR Cycles
***COL1***	AF129287.1	F: CGTGATCTGCGACGAACTTAA	212	40
	Ovine	R: GTCCAGGAAGTCCAGGTTGT		
***COL3***	AY091605.1	F: AAGGGCAGGGAACAACTTGAT	355	40
	Ovine	R: GTGGGCAAACTGCACAACATT		
***TNMD***	NM_001099948.1	F: TGGTGAAGACCTTCACTTTCC	352	40
	Bos Taurus	R: TTAAACCCTCCCCAGCATGC		
***THBS4***	NM_001034728.1	F: CCGCAGGTCTTTGACCTTCT	231	40
	Bos Taurus	R: CAGGTAACGGAGGATGGCTTT		
***SCXB***	XM_866422.2	F: AACAGCGTGAACACGGCTTTC	299	45
	Bos Taurus	R: TTTCTCTGGTTGCTGAGGCAG		
***OCN***	DQ418490.1	F: AGACACCATGAGAACCCCCAT	234	40
	Ovine	R: TTGAGCTCACACACCTCCCT		
***GAPDH***	AF030943.1	F: CCTGCACCACCAACTGCTTG	224	40
	Ovine	R: TTGAGCTCAGGGATGACCTTG		

In particular:

*SCXB*, a bHLH transcription factor gene a crucial marker for the tendon cell fate [38], [71] and embryogenetic tendon differentiation marker [71]–[73],
*COL1* and *3*, the main components of extracellular matrix,
*TNMD*, tendon transmembrane glycoproteins [74],
*THBS4*, extracellular matrix protein,
*OCN*, a generic mesenchymal derived tissue related protein.

In detail, total mRNA was extracted by using TRI Reagent (Sigma) according to the manufacturer's instructions. Integrity and size distribution were evaluated by 1% agarose gel electrophoresis and ethidium bromide staining. Digestion of genomic DNA was carried out by DNaseI digestion (Sigma) for 15 minutes at RT. 1 µg of total RNA of each sample was used for reverse transcription reaction with Oligo dT primer and BioScriptTM Kit (Bioline). 2× Ready mix™ Taq PCR Reaction mix (Sigma) was used for PCR reaction using 3 µl of cDNA and 0.5 µM of each primer, in a final volume of 25 µl. The primer sequences, Genebank number of reference mRNA sequence, product length and cycles are shown in Table 4. The reaction mixtures were incubated for 5 minutes at 95°C, followed by 95°C for 30 seconds, 55°C for 30 seconds, 72°C for 45 seconds and 72°C for 7 minutes. For each gene, a reaction mixture with water instead of cDNA template was run at the same time as a PCR negative control. RT-PCR was normalized by the transcriptional levels of *GAPDH*. The PCR products were separated on 2% agarose gel stained with ethidium bromide, visualized on a Gel Doc 2000 (Biorad) and analyzed with Quantity One 1-D Analysis software (Biorad). Each PCR reaction was carried out in triplicate.

### Statistical analysis

Data reported in this paper are the mean (±SD) of at least 3 independent experiments, each performed in triplicate. The data were checked for parametric distribution by Shapiro-Wilks W test, before comparing them with ANOVA test followed, if necessary, by *post-hoc* Tukey test (StatistiKL Version β). The differences were considered significant and highly significant for *p*<0.05 and <0.01, respectively.
